# Effect of selected local medicinal plants on the asexual blood stage of chloroquine resistant *Plasmodium falciparum*

**DOI:** 10.1186/1472-6882-14-492

**Published:** 2014-12-15

**Authors:** Mohd Ridzuan Mohd Abd Razak, Adlin Afzan, Rosnani Ali, Nur Fasihah Amir Jalaluddin, Mohd Isa Wasiman, Siti Habsah Shiekh Zahari, Noor Rain Abdullah, Zakiah Ismail

**Affiliations:** Herbal Medicine Research Centre, Institute for Medical Research, Jalan Pahang, 50588 Kuala Lumpur, Malaysia

## Abstract

**Background:**

The development of resistant to current antimalarial drugs is a major challenge in achieving malaria elimination status in many countries. Therefore there is a need for new antimalarial drugs. Medicinal plants have always been the major source for the search of new antimalarial drugs. The aim of this study was to screen selected Malaysian medicinal plants for their antiplasmodial properties.

**Methods:**

Each part of the plants were processed, defatted by hexane and sequentially extracted with dichloromethane, methanol and water. The antiplasmodial activities of 54 plant extracts from 14 species were determined by *Plasmodium falciparum* Histidine Rich Protein II ELISA technique. In order to determine the selectivity index (SI), all plant extracts demonstrating a good antiplasmodial activity were tested for their cytotoxicity activity against normal Madin-Darby Bovine Kidney (MDBK) cell lines by 3-(4, 5-Dimethylthiazol-2-yl)-2, 5-diphenyltetrazolium bromide (MTT) assay.

**Results:**

Twenty three extracts derived from *Curcuma zedoaria* (rhizome), *Curcuma aeruginosa* (rhizome), *Alpinia galanga* (rhizome), *Morinda elliptica* (leaf), *Curcuma mangga* (rhizome), *Elephantopus scaber* (leaf), *Vitex negundo* (leaf), *Brucea javanica* (leaf, root and seed), *Annona muricata* (leaf), *Cinnamomun iners* (leaf) and *Vernonia amygdalina* (leaf) showed promising antiplasmodial activities against the blood stage chloroquine resistant *P. falciparum* (EC_50_ < 10 μg/ml) with negligible toxicity effect to MDBK cells *in vitro* (SI ≥10).

**Conclusion:**

The extracts belonging to eleven plant species were able to perturb the growth of chloroquine resistant *P. falciparum* effectively. The findings justified the bioassay guided fractionation on these plants for the search of potent antimalarial compounds or formulation of standardized extracts which may enhance the antimalarial effect *in vitro* and *in vivo*.

## Background

Malaria is one of the major public health problems in many tropical regions, including Malaysia. The resistance *Plasmodium falciparum* to common antimalarial drugs such as chloroquine, sulfadoxine-pyrimethamine [1–3] and artemisinin [4] have been reported. In response to this situation, as recommended by World Health Organization (WHO), Malaysian government has changed its first line antimalarial drug regimen to artemisinin-based combination therapy (ACT) such as the use of fixed dose artemether-lumefantrine combination (Riamet®) in the treatment of uncomplicated falciparum malaria (National Antibiotic Guideline 2008, Ministry of Health, Malaysia). However, the ability of the malaria parasite such as *P. falciparum* to develop and become resistant to ACT in the future cannot be denied [5–9]. Therefore, discovering new antimalarial drugs is a priority in the health sector. The challenges in malaria drug discovery are to find safe, cheap and effective antimalarial agents.

Plants have always been the main source for the search of new antimalarial drugs. Until the year of 2003, 1277 plant species from 160 families have been published by 33 tropical countries for their use in treatment of malaria and fevers [10]. In Peninsular Malaysia, about 21 plant species are used by the locals as traditional medicine for malaria treatment [11–13]. So, it is of pivotal to know the potential ingredients or candidates which play a major role in killing the malaria parasites. Thus, screening the plant extracts for antimalarial properties prior to bioassay guided fractionation and potent compound isolation is important.

Research on the effectiveness of medicinal plant extracts in inhibiting the growth of malaria parasite has been extensively studied worldwide. One good example is *Artemisia annua* where whole leaves extract of this plant has exhibited better antiplasmodial activity as compared to its isolated compound, artemisinin [14, 15]. This shows that there are other unidentified compounds still remain in this plant.

Many of medicinal plants which grow in Malaysian soil have been reported by the local scientists for their antiplasmodial activities *in vitro* and *in vivo* (Table 1). In this study, another 14 selected Malaysian medicinal plants (Table 2) with traditional claims were screened for their antiplasmodial activity against the malaria parasite, chloroquine (CQ) resistant *P. falciparum* (K1) *in vitro* by using *P. falciparum* Histidine Rich Protein II (HRP2) ELISA technique [16].

**Table 1:** **The list of Malaysian medicinal plants with potential antimalarial properties**

Plant species	Local name	Plant part	References
*Agathis borneensis*	Raja Kayu	Leaf	[17]
*Alpinia galanga*	Lengkuas	Rhizome	[18]
*Alstonia angustiloba*	Akar Lumut	Leaf	[19]
*Alyxia lucida*	Mempelas Hari	Leaf	[20]
*Andrographis paniculata*	Hempedu Bumi	Leaf	[20–22]
*Ardisia crenata*	Mata Ayam	Root	[21]
*Ardisia crispa*	Mata Itik	Leaf	[17, 20]
*Blumea balsamifera*	Sembong	Root, Stem	[17]
*Calotropis gigantea*	Rembega	Leaf	[19]
*Carica papaya*	Betik	Leaf	[21]
*Cinnamomum iners*	Teja Lawang	Root	[21]
*Cocos nucifera*	Kelapa	White flesh	[23]
*Croton argyratum*	Semangkok	Leaf	[17]
*Cryptocarya nigra*	Medang	Stem	[24]
*Dyera costulata*	Jelutong	Leaf	[19]
*Eurycoma longifolia*	Tongkat Ali	Root	[25–27]
*Goniothalamus macrophyllus*	Selada	Stem	[17]
*Goniothalamus scorthechinii*	Selada Putih	Root, Stem, Leaf	[17, 22]
*Gynura procumbens*	Sambung Nyawa	Leaf	[28]
*Jasminum sambac*	Melati	Flower	[21]
*Kopsia fruticosa*	Chabai Hutan	Leaf	[19]
*Lansium domesticum*	Langsat	Leaf, fruit skin	[29]
*Leuconotis eugenifolius*	Cheret Murai	Bark	[30]
*Macaranga triloba*	Mahang Merah	Inflorescence	[31]
*Nigella sativa*	Jintan Hitam	Seed	[21]
*Ocimum sanctum*	Selasih	Whole plant	[21]
*Phoebe grandis*	Medang	Leaf	[32]
*Physalis minima*	Letup-letup	Whole plant	[21]
*Piper betle*	Sireh	Leaf	[33]
*Piper sarmentosum*	Kaduk	Leaf	[34]
*Rennellia elliptica*	Segemuk	Root	[35]
*Tinospora crispa*	Patawali	Stem	[21]
*Vallaris glabra*	Kerak nasi	Leaf	[19]
*Xylocarpus granatum*	Nyireh	Bark	[21]

Extracts of these plants have been reported for their antiplasmodial (*in vitro*) and/or antimalarial (*in vivo*) activities by the Malaysian researchers from year 1995 to 2013.

**Table 2:** **The list of 14 Malaysian medicinal plants and its traditional claims and treatments**

Family	Plant species	Local name	Traditional claims by the Malays and aborigines	Voucher specimen
*Asteraceae*	*Vernonia amygdalina*	Pokok panjang hayat, Daun bismillah	Leaf: Used as a remedy for the management of diabetes, hypertension and hypercholestrolaemia.	PID231114-18
*Simaroubaceae*	*Brucea javanica*	Melada pahit	Leaf: Used as a poultice for scurf, ringworm, boils, centipede bites and over enlarged spleen in fever. Root: Used as a decoction for colic, dysentery, fever, bodily pain and labour pain. Fruit and leaf: Used as an infusion to cure malaria.	UKMB40227
*Leeaceae*	*Leea indica*	Mali-mali, memali	Leaf: Used as a poultice in skin complaints caused by poisonous caterpillars and body pains.	PID241114-18
*Lauraceae*	*Cinnamomun iners*	Kayu manis hutan, Teja lawang	Root: Used as a decoction after childbirth, fever. Leaf: Used as a poultice for rheumatism.	PID271114-18
*Verbenaceae*	*Vitex negundo*	Lenggundi, Lemuni hitam	Leaf: Used as a remedy for cleansing the birth canal and increase the production of milk after childbirth.	PID261114-18
*Combretaceae*	*Terminalia catappa*	Ketapang	Bark: Act as astringent in dysentery Leaf: Act as a sudorific and applied to rheumatic joints, used internally for headache and colic.	PID251114-18
*Rubiaceae*	*Morinda elliptica*	Mengkudu kecil, Mengkudu hutan	Leaf: Added to rice for loss of appetite, taken for head ache, cholera, diarrhoea and fever. Applied in a pounded condition upon the spleen and wounds. A lotion for haemorrhoids and upon the body after childbirth.	MTM193
*Annonaceae*	*Annona muricata*	Durian belanda	Leaf: Used as a poultice or an infusion externally for skin complaints in children, and for coughs and rheumatism.	MTA174
*Asteraceae*	*Elephantopus scaber*	Tutup bumi, Tapak sulaiman	Used as a decoction (leaf or root) for preventive medicine after childbirth, in tonics, to drive out round worm, for coughs and veneral disease. The leaf decoction used as an antihelmintic, as a diuretic and for abdominal pains. The root decoction also used to arrest vomiting.	MTE174
*Zingiberaceae*	*Curcuma mangga*	Temu pauh, Temu manga	Rhizome: Used as stomachic and as a mixture for continuous fever.	RZ14/10
	*Curcuma zedoaria*	Temu kuning, Temu putih	Rhizome: Used in decoction as a tonic and for indigestion.	MTC0071
	*Curcuma aeruginosa*	Temu hitam, Temu erang	Rhizome: Used as a tonic, for a cough and asthma. Externally used (pounded in coconut oil) for scurf.	RZ18/10
	*Alpinia galanga*	Lengkuas	Rhizome: Used as a decoction to cure malaria. Carminative, stomachic and ointment for skin eruptions.	MTA0059
	*Curcuma phaeocaulis*	Temu merah	Rhizome: Used by the local to treat tumours	RZ19/10

As referred in [11, 36] and Global Information Hub in Integrated Medicine (http://www.globinmed.com).

## Methods

### Plant collection and identification

All plant parts except *Brucea javanica* and *Annona muricata* were collected from the herbal garden of Herbal Medicine Research Centre (HMRC), Institute for Medical Research (IMR), Kuala Lumpur, Malaysia*.* Both *B. javanica* and *A. muricata* were collected from Northern part of Peninsular Malaysia, Tupah Village, Kedah andMalaysian Agricultural Research and Development Institute (MARDI), Kedah, respectively. All plants parts except *B. javanica*, *Curcuma aeruginosa*, *C. mangga, C. phaeocaulis* were identified and authenticated by Dr. Richard Chung Cheng Kong and deposited in the Herbarium of Forest Research Institute Malaysia (FRIM), Kepong, Kuala Lumpur. The *B. javanica* was identified and authenticated by Mr. Sani Miran and deposited in the Herbarium of the Universiti Kebangsaan Malaysia (UKM), Bangi, Selangor, Malaysia. The *Curcuma* species were identified and deposited in the Herbarium of Herbal Medicine Research Centre, Institute for Medical Research, Kuala Lumpur, Malaysia.

### Plant extract preparation

**Figure 1:**
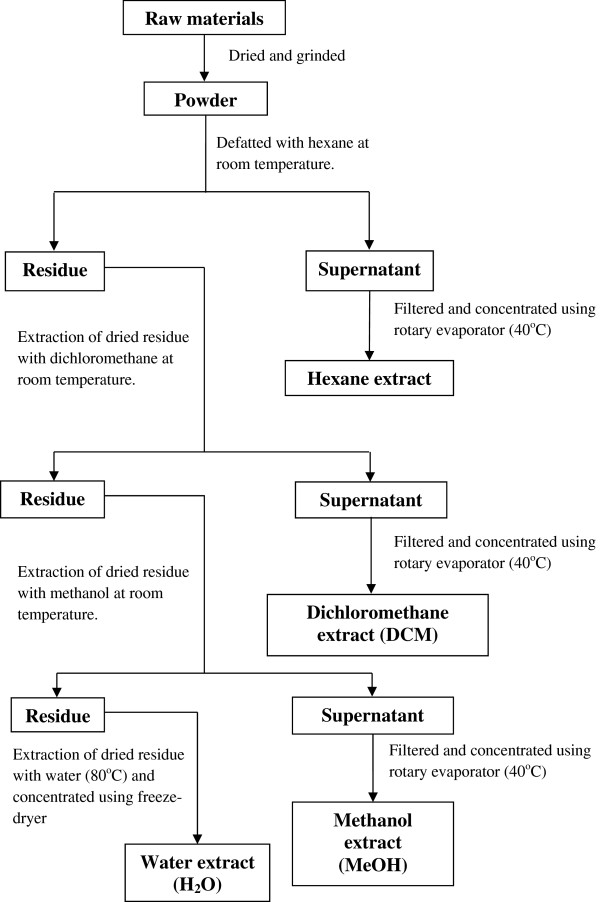
**Procedure of plant extraction.** Different part of plants was extracted sequentially by using organic solvents (DCM and MeOH) with increasing polarity.

The fresh plant materials (rhizome, leaf, root and seed) were cut into small pieces, dried and pulverized into powder before extraction with solvents in increasing polarity (Figure 1). The powdered materials were first defatted with hexane and sequentially extracted with dichloromethane (DCM), methanol (MeOH) and sterile deionised water (H_2_O) (80°C). Briefly, the resulting solutions from the first extraction using DCM were filtered through filter paper (Whatman No.1, England) to collect the supernatant from the residue. Organic supernatant were evaporated to dryness under reduced pressure with a rotary evaporator (Buchi Rotavapor R-200, Switzerland) at a temperature 40°C. The residue was further extracted by using MeOH similar to the procedure that carried out for the DCM. The resulting residue was air dried and used for further extraction with sterile H_2_O at 80°C. The aqueous supernatant were freeze-dried to obtain crude extracts (Figure 1). All crude extracts (DCM, MeOH and H_2_O) were stored at 4°C until used.

### The mammalian cells and parasites

The CQ resistant *P. falciparum* strain, K1 was obtained from American Type Culture Collection (ATCC), The Malaria Research and Reference Reagent Resource Center (MR4). Madin-Darby Bovine Kidney (MDBK) cells were also obtained from ATCC. The cryopreserved parasites and cells were thawed and maintained in culture for further use in this study. Briefly, the cryopreserved parasites were thawed in waterbath at 37°C. The thawed parasites were transferred into 15 ml conical tubes and equal volume of 3.5% natrium chloride (NaCl) were added drop wise and swirled to mix. The mixtures were centrifuged at 500 × g for 5 minutes and the supernatants were discarded. These steps were repeated twice before adding the complete RPMI 1640 culture medium (Invitrogen, USA) containing 25 mM 4-(2-hydroxyethyl)-1-piperazineethanesulfonic acid (HEPES), 0.2% sodium bicarbonate (NaHCO_3_), 0.02 mg/ml gentamycin supplemented with 10% AB+ human serum (Invitrogen, USA).

For MDBK cells, the cryopreserved cells were thawed immediately in the waterbath at 37°C. The thawed cells were transferred in 15 ml conical tubes containing 1 ml of complete DMEM culture medium (Invitrogen, USA) containing 25 mM HEPES, 0.4% sodium bicarbonate (NaHCO_3_), 100U of Penstrep (100U penicillin and 100U streptomycin) supplemented with 10% fetal bovine serum (FBS). The mixtures were centrifuged at 1000 × g, 4°C for 5 minutes. The supernatants were discarded. The cell pellets were transferred into 25 cm^2^ culture flask containing 5 ml of complete DMEM culture medium. The suspension were mixed gently and incubated in 5% carbon dioxide (CO_2_) incubator at 37°C.

### *In vitro*culture and synchronization of *P. falciparum*

The CQ resistant *P. falciparum* were grown by candle jar technique (3% CO_2_ and 17% O_2_) [37]. The culture was set up in a 25 cm^3^ culture flask with filtered vent and maintained in complete RPMI 1640 culture medium (Invitrogen, USA). The *P. falciparum* was grown in ‘O’ type fresh red blood cell (RBC) with the initial culture started with 1% parasitemia at 2.5% hematocrit. The parasite density was monitored daily by making thin blood smears stained with 10% Giemsa solution and observed under the microscope at 1000 times magnification. When the parasitemia of the parasite culture reached approximately 5 to 7%, the parasites were synchronized using 5% sorbitol [38] and cultured for one complete cycle prior to be used in *in vitro P. falciparum* HRP2 assay.

### *In vitro P. falciparum*HRP2 assay

All crude extracts were evaluated *in vitro* for their antiplasmodial activities by HRP2 assay [16, 39]. The extracts were solubilised in 100% dimethyl sulphoxide (DMSO) or sterile H_2_O (H_2_O extracts only) to get 5 mg/ml stocks. In preparation of extract or drug stock plates, the extracts (5 mg/ml) were serially diluted (2 fold dilution) to 7 point concentrations (ranging from 5 to 0.08 mg/ml) in DMSO from well A1 to A7 in a 96 well plate. Fifteen microliters of serially diluted stock extracts were transferred correspondingly into watery plates containing 225 μl of sterile H_2_O. An aliquote of watery plates will be used in HRP2 assay.

Ring-infected RBCs with 5% parasitemia were adjusted to 0.05% parasitemia and 1.5% hematocrit. A total of 190 μl parasitized RBCs at 1.5% hematocrit were added into each well of the test plates. A total of 10 μl of serially diluted extracts from the watery plates prepared above were transferred into the test plates containing parasitized RBCs and incubated in a candle jar at 37°C for 72 hours. The final tested concentration ranging from 16 to 0.2 μg/ml. The final concentration of DMSO was 0.3%. Chloroquine (CQ) (Sigma, USA), quinine (Q) (Sigma, USA), mefloquine (Mef) (Sigma, USA) and artemisinine (Art) (Sigma, USA) were used as standard control to validate the test. The final tested concentration for standard control ranged from 1772.6 to 27.7 nM for CQ, 3495 to 54.6 nM for Q, 601.3 to 9.4 nM for Mef and 51.2 to 0.8 nM for Art. The negative control was the infected RBC without extracts or with sterile H_2_O only. After 72 hours of incubation, the test plates were kept in -80°C overnight. The plates were thawed at room temperature to lyse the infected RBCs. The activity of the parasite-extract exposure (end point) was measured by HRP2 assay. One day prior to the assay, 100 μl of immunoglobulin M (IgM) capture antibody (MPFM-55A, ICL, Inc, Newberg, OR, USA) specific for *P. falciparum* HRP2 (1 μg/ml in phosphate-buffered saline (PBS)) were added to each well of a 96-well ELISA plates (Microlon 600, Greiner, Germany). The plates were covered and incubated at 4°C overnight. Following incubation, the contents of the wells were removed and the plates were washed three times with 0.05% PBS-Tween 20 (PBST). The non-binding sites of the ELISA plates were blocked with 200 μl/well of 2% bovine serum albumin in PBS for 2 hours at room temperature. Following the blocking step, the ELISA plates were washed 3 times with 200 μl of 0.05% PBST. Hundred microliters of the *P. falciparum* infected RBC lysates (freeze thawed) were transferred from the test plates into ELISA plates and incubated in humidity chamber for 1 hour at room temperature. The ELISA plates were washed as described above. Hundred microliters of the detector antibody (MPFG-55P, ICL, Inc, Newberg, OR, USA) conjugated with horseradish peroxidase (0.2 μg/ml in PBS) were added to each well, and incubated in humid chamber for 1 hour at room temperature. Following a subsequent washing step similar to the above, 100 μl of 3,3’, 5,5;-tetramethylbenzidine (TMB) chromogen (Zymed Lab., Inc., San Francisco, CA, USA) was added to each well and incubated for 10 min in dark, followed by the addition of 50 μl of 1 M sulphuric acid. The absorbance was determined by using ELISA plate reader at a wavelength of 450 nm (FLUOstar Omega, Germany). The collected data were transferred to HN-nonLin software (malaria.farch.net) to get a 50% Effective Concentration (EC_50_) value directly from the graph.

### *In vitro*cytotoxicity assay

The MDBK cells were maintained in complete DMEM culture medium containing 25 mM HEPES, 0.4% sodium bicarbonate (NaHCO_3_), 100U of Penstrep (100U penicillin and 100U streptomycin) supplemented with 10% fetal bovine serum (FBS). The cytotoxicity of the extracts were measured by 3-(4, 5-Dimethylthiazol-2-yl)-2, 5-diphenyltetrazolium bromide (MTT) assay [40]. Prior to the day of test, the stock plates were prepared by serially diluting (2 fold dilution) the stock extracts (5 mg/ml) to 7 point concentration (ranging from 5 to 0.16 mg/ml) with DMSO or sterile H_2_O (for H_2_O extracts only). Then, 6 μl of serially diluted stocks were transferred into 96 well plates containing 294 μl of complete DMEM media (Medium plates). On the day of the test, MDBK cells were harvested and adjusted to 1 × 10^4^ cell per ml. A hundred microliter of cell suspension was seeded into each well of a 96-well plate and allowed to grow overnight. Then, 100 μl of test extracts taken from medium plate (as prepared above) were added to each well accordingly ranging final concentration of 0.8 to 50 μg/ml. The final concentration of DMSO in all test was less than 1%. All tests were performed in duplicate. The positive control for cell growth is the cell suspension without test substance while the negative control is the cell suspension with 0.05% Triton X 100. The culture was incubated at 37°C in 5% CO_2_ incubator for 72 hours. Fifty microliters of MTT solution (5 mg MTT in 1 ml PBS and 2.5 ml DMEM media) were added to each well. The plates were further incubated for 4 hours at 37°C in 5% CO_2_ incubator. The medium was removed and replaced with 200 μl of DMSO to solubilise the MTT formazan product. The solution was mixed for 15 min and once for 30 sec before measuring the absorbance at 540 nm with a micro plate reader (FLUOstar Omega, Germany). The percentage of growth inhibition and the EC_50_ were estimated from a dose response curve.

### Determination of a selectivity index

A selectivity index (SI), corresponding to the ratio between antiplasmodial and cytotoxic activities was calculated according to the following formula:


## Results

### Antiplasmodial activity against CQ resistant *P. falciparum*, K1 strain

A total of 54 extracts from different parts of 14 plant species (Table 2) were tested for antiplasmodial activity against chloroquine resistant *P. falciparum* by using HRP2 assay *in vitro*. Each part of the plants was extracted by 3 different solvents with increasing polarity (DCM-MeOH-H_2_O). The discrimination of active extracts is based on ranked levels of antiplasmodial activity proposed by Rasoanaivo et al. [41]. The score for the test is classified as extracts with EC_50_ value less than 0.1 μg/ml is considered to be very good, 0.1 to 1.0 μg/ml is good, 1.1 to 10 μg/ml is good to moderate, 11 to 25 μg/ml is weak, 26 to 50 μg/ml is very weak while more than 100 μg/ml is considered inactive [41]. So, any extracts which exhibit an EC_50_ value less than 10 μg/ml (EC_50_ ≤ 10 μg/ml) is considered to have potential or promising antiplasmodial activity. The antiplasmodial HRP2 assay on the plant extracts were performed in parallel with the standard antimalarial drugs such as CQ, Q, Mef and Art which act as a control for the validity of the assay (Table 3). Briefly, the results of each assay were validated by determination of EC_50_ values produced by the standard antimalarial drugs against CQ resistant *P. falciparum*. In this study, the assay is considered valid when the EC_50_ value of CQ is more than 100 nM [42].

**Table 3:** **Representative of** ***in vitro*** **antiplasmodial activity of standard drugs against** ***P. falciparum*** **K1 strain**

Drugs	EC _50_ values	
	nM	μg/ml
Chloroquine	149.43	0.077
Quinine	152.19	0.049
Mefloquine	23.49	0.010
Artemisinin	1.80	0.00051

Data are presented as mean of three independent experiments performed in duplicate assays.

The *in vitro P. falciparum* HRP2 assay for standard antimalarial drugs were conducted in parallel with the test plant extracts. Each of the assay were validated based on the EC_50_ threshold for each standard antimalarial drugs against the *P. falciparum* K1 strain in the lab. If the EC_50_ value of the standard antimalarial drugs are out of range, the test considered invalid.

The DCM, MeOH and H_2_O extracts from 14 plants species showed wide range of antiplasmodial activities (Table 4). Overall 41 extracts (76%) from 13 plant species showed promising antiplasmodial activity (EC_50_ < 10 μg/ml) (Table 5). In detail, there are 11 extracts from 5 plant species fall within a good level of antiplasmodial activity (EC_50_ < 1 μg/ml) (Table 5). Good to moderate antiplasmodial activities (EC_50_ = 1.1-10 μg/ml) were detected in 30 extracts from 12 plant species (Table 5). Thirteen extracts from 6 plant species were considered weak or inactive as these extracts exhibited EC_50_ values of more than 10 μg/ml or less than 50% parasite inhibition at the highest tested concentration (EC_50_ > 15.7 μg/ml) (Table 5). Overall, DCM and MeOH extracts were more active against CQ resistant *P. falciparum* with good level of antiplasmodial activity (Table 5).

**Table 4:** **The antiplasmodial and cytotoxicity activities of DCM, MeOH and H**_**2**_ **O extracts of selected Malaysian medicinal plants**

Plant name	Parts	Extracts	EC _50_(μg/ml)		Selectivity index (SI)
			***P. falciparum***K1	MDBK cells	
*Curcuma zedoaria*	Rhizomes	DCM	2.38	37.61	15.92
		MeOH	>15.70	NT	ND
		H_2_O	>15.70	NT	ND
*Vernonia amygdalina*	Leaves	DCM	3.36	157.69	47.00
		MeOH	1.09	>300	>274.39
		H_2_O	8.23	>600	>72.95
*Alpinia galanga*	Rhizomes	DCM	1.64	3.92	2.40
		MeOH	8.06	87.95	10.92
		H_2_O	1.84	>50	>27.16
*Brucea javanica*	Leaves	DCM	0.55	15.13	27.64
		MeOH	5.27	87.95	16.70
		H_2_O	1.29	1.90	1.48
	Roots	DCM	0.47	6.54	14.05
		MeOH	0.58	6.95	12.06
		H_2_O	4.49	9.59	2.14
	Green seeds	DCM	>15.70	NT	ND
		MeOH	0.25	NT	ND
		H_2_O	1.47	NT	ND
	Black seeds	DCM	>15.70	NT	ND
		MeOH	1.96	7.70	3.92
		H_2_O	5.40	24.24	4.49
*Leea indica*	Leaves	DCM	>15.70	NT	ND
		MeOH	>15.70	NT	ND
		H_2_O	>15.70	NT	ND
*Cinnamomun iners*	Leaves	DCM	1.95	199.15	102.58
		MeOH	0.63	>400	>636.61
		H_2_O	0.62	>100	>161.29
*Vitex negundo*	Leaves	DCM	1.10	25.04	22.80
		MeOH	2.17	>50	>23.10
		H_2_O	>15.70	NT	ND
*Terminalia catappa*	Leaves	DCM	5.29	>50	>9.45^a^
		MeOH	5.19	>50	>9.63^a^
		H_2_O	4.28	25.02	5.84
*Morinda elliptica*	Leaves	DCM	9.08	>50	>5.51^a^
		MeOH	7.76	>50	>6.45^a^
		H_2_O	0.50	31.05	62.10
*Annona muricata*	Leaves	DCM	0.61	40.48	66.47
		MeOH	0.26	>100	>387.60
		H_2_O	0.27	>200	>756.14
*Curcuma mangga*	Rhizomes	DCM	6.59	36.66	4.09
		MeOH	1.81	>50	15.44
		H_2_O	2.30	31.85	11.27
*Elephantopus scaber*	Leaves	DCM	3.71	8.89	11.27
		MeOH	0.27	42.75	2.39
		H_2_O	>15.70	NT	ND
	Roots	DCM	>15.70	NT	ND
		MeOH	>15.70	NT	ND
		H_2_O	>15.70	NT	ND
*Curcuma aeruginosa*	Rhizomes	DCM	5.40	>50	>9.26^a^
		MeOH	5.32	>50	>9.39^a^
		H_2_O	4.78	>50	>10.46
*Curcuma phaeocaulis*	Rhizomes	DCM	5.00	11.04	2.21
		MeOH	9.24	>50	>5.41^a^
		H_2_O	10.45	NT	ND

Data are presented as mean of at least two independent experiments performed in duplicate assays. DCM, dichloromethane; MeOH, methanol; H_2_O, water extracts. No activity at the highest tested concentration denoted as EC_50_ > 15.70 μg/ml. No toxicity at the highest tested concentration denoted as EC_50_ > 50.00, 100, 200, 300, 400 and 600 μg/ml. SI: EC_50 MDBK cell lines_ / EC_50 *Plasmodium*_. ^a^The SI is not conclusive because the EC_50_ value is higher than 50 μg/ml which might resulted in higher SI value. NT, not tested, ND, not determined.

*In vitro P. falciparum* HRP2 and MTT assays were conducted for each plant extracts on CQ resistant *P. falciparum* and MDBK cell line, respectively. The EC_50_ value for each plants extracts were determined from the dose-response curves produced by using the HN-nonLin software (malaria.farch.net). The ratio of MDBK cell line EC_50_ and *P. falciparum* EC_50_ of each extract give the SI index or the level of toxic effects. Twenty three extracts from 11 plant species showed promising antiplasmodial activity (EC_50_ ≤ 10 μg/ml) with no toxic effect (SI ≥ 10).

**Table 5:** **The summary of antiplasmodial level of extracts from different parts of 14 plant species**

Antiplasmodial level	Plant species	Parts	Extracts
**Good** EC_50_ = 0.1-1.0 μg/ml	*A. muricata*	Leaves	DCM, MeOH, H_2_O
	*B. javanica*	Green seeds	MeOH
		Roots	DCM, MeOH
		Leaves	DCM
	*E. scaber*	Leaves	MeOH
	*M. elliptica*	Leaves	H_2_O
	*C. iners*	Leaves	H_2_O, MeOH
**Good to moderate** EC_50_ = 1.1- 10 μg/ml	*V. amygdalina*	Leaves	DCM, MeOH and H_2_O
	*A. galanga*	Rhizomes	DCM, MeOH and H_2_O
	*T. catappa*	Leaves	DCM, MeOH and H_2_O
	*C. mangga*	Rhizomes	DCM, MeOH and H_2_O
	*C. aeruginosa*	Rhizomes	DCM, MeOH and H_2_O
	*C. zedoaria*	Rhizomes	DCM
	*E. scaber*	Leaves	DCM
	*B. javanica*	Leaves	MeOH and H_2_O
		Roots	H_2_O
		Green seeds	H_2_O
		Black seeds	MeOH and H_2_O
	*C. iners*	Leaves	DCM
	*V. neguno*	Leaves	DCM and MeOH
	*M. elliptica*	Leaves	DCM and MeOH
	*C. phaeocaulis*	Rhizomes	DCM and MeOH
**Weak** EC_50_ = 11-25 μg/ml	*C. phaeocaulis*	Rhizomes	H_2_O
	*B. javanica*	Green seeds	DCM
		Black seeds	DCM
	*L. indica*	Leaves	DCM, MeOH and H_2_O
	*C. zedoaria*	Rhizomes	MeOH and H_2_O
	*V. negundo*	Leaves	H_2_O
	*E. scaber*	Leaves	H_2_O
		Roots	DCM, MeOH and H_2_O

The level of efficacy of extracts were ranked according EC_50_ values which based on the threshold for *in vitro* antiplasmodial activity proposed by Rasoanaivo et al. [41]. Forty one extracts from 13 plant species were categorized to have good and good to moderate antiplasmodial level.

### Cytotoxicity activity to mammalian MDBK cell line

Forty one extracts from 13 different plant species with promising antiplasmodial activity (EC_50_ < 10.0 μg/ml), were subjected to MTT cytotoxicity assay and tested against the MDBK cell lines (Table 4). In fact, the pharmacological efficacy of the extracts is considered selective and nontoxic when SI is ≥10 [43]. Twenty three of antiplasmodial plant extracts (DCM, MeOH or H_2_O) from 11 plant species showed a specific selectivity (SI >10) towards chloroquine resistant *P. falciparum*, K1 rather than MDBK cells with SI ranging from 10.46 to 756.14 (Table 4). The extract from *B. javanica* green seed was not tested for MTT assay and has been excluded from this experiment.

## Discussion

The present study has identified the antiplasmodial activity in selected plant extracts by HRP2-based assay or HRP2 ELISA technique. There are other reports on antiplasmodial studies using different screening methods such as WHO schizont maturation test [44], isotopic assay [45], pLDH enzymatic assay [46, 47], SYBR Green I assay [48–50] or fluocytometric assay [51]. Briefly, the HRP2-based assay is a very sensitive and specific measures of *P. falciparum* growth by quantifying parasite specific biomolecule, HRP2. The suitability, reproducibility and sensitivity of HRP2-based assay in antimalarial drug screening is well documented since year 2002 [16, 39, 52–55]. The HRP2-based assay is comparable with other techniques because the result produced by this assay has been previously shown to be closely parallel those obtained from the isotopic assay, traditional WHO schizont maturation tests [54] and SYBR green I assay [56].

Eleven plants (79%) were identified to possess promising antimalarial properties in at least one of their extracts (DCM, MeOH or H_2_O). These findings are based on their potent antiplasmodial activities (EC_50_ ≤ 10 μg/ml) and high preferences in killing the malaria parasite rather than mammalian cell line (SI ≥10) (Table 4). Most of the potential antiplasmodial activity were exhibited by DCM and MeOH extracts which may be related to the presence of alkaloids, terpenoids and flavonoids [57, 58]. Theoretically, the purpose of using this extraction technique is to extract specific classes of phytochemical constituents from non-polar compounds to polar compounds [59]. The crude extract of DCM usually contains intermediate polarity of compounds such as alkaloids, steroids and terpenoids [59–61]. These classes of compounds especially alkaloids are well known as active constituents against antiplasmodial activity. In fact, one of the oldest and most known antimalarial drug, quinine belongs to this class of compounds. In addition, an example of common terpenoids is artemisinin, the most potent antimalarial to date [58]. Extraction with methanol will extract more polar compounds such as flavonoid glycosides, saponin, tannins and anthocyanins [59–61]. The antimalarial activity from these classes of compounds especially flavonoids have been described earlier [57]. Further extraction with water will extract high polarity of compounds such as phenolic acids, sugars and glycosides [59–61].

In another point of view, majority of these plants also possessed at least 1% of CQ antiplasmodial activity indicating the potential of these plants to be the source of antimalarial candidates. For example, the ethanolic extract of *Artemisia annua* Linn leaves (the source of artemisinin) inhibited the growth of CQ resistant (K1) and CQ sensitive (3D7) strains of *P. falciparum* with IC_50_ of 10.4 μg/ml and 21.8 μg/ml, respectively [49]. So, even the plant like *A. annua* with weak antiplasmodial activity (only 0.007% and 0.004% of artesunate activity against *P. falciparum* K1 and 3D7, respectively) contains the most potent antimalarial compound to date. This phenomenon may also apply to the plants extracted in this study.

In this study, *A. galanga* (rhizome), *C. iners* (leaf), *C. zedoaria* (rhizome), *E. scaber* (leaf), *C. mangga* (rhizome) and *M. elliptica* (leaf) were for the first time reported for their good level of antiplasmodial activities *in vitro*. Other potent antiplasmodial plants such as *C. aeruginosa* (rhizome) [62], *V. negundo* (leaf) [46], *B. javanica* (leaf and root) [45, 51, 62], *A. muricata* (leaf) [63] and *V. amygdalina* (leaf) [44, 64–68] have been widely studied and were further discussed in this section.

### A. galanga

The *A. galanga* has been identified as one of the plants traditionally used in some part of Peninsular Malaysia [11]. The *in vitro* antiplasmodial data of *A. galanga* rhizome extracts (EC_50_ < 10 μg/ml) reported by present study is complementing the *in vivo* study conducted by Al-Adhroey et al. [11]. The MeOH extract of the rhizome exhibited a significant suppressive, curative and prophylactic activities on *P. berghei* infected mice. The antimalarial properties of MeOH extract of *A. galanga* rhizome could be governed by its active constituents such as flavonoids and terpenoids [18]. In addition, both terpenoids and flavonoids related compounds have been previously shown to exhibit antiplasmodial activities against several *P. falciparum* strains [57].

### E. scaber

In this study, the good level of antiplasmodial activity showed by the MeOH extract of *E. scaber* leaf (EC_50_ = 0.27 μg/ml) was contradictory to the study reported by Kantamreddi and Wright [46]. According to Kantamreddi and Wright [46], the MeOH extract of the leaves of this plant was considered inactive (IC_50_ = 133.8 μg/ml) against CQ resistant *P. falciparum* (K1) [46]. The possible reason for the contradictory results could be due to the differences in the duration of incubation in antiplasmodial assay. The incubation period for the present study is longer (72 hours) than the Kantamreddi and Wright [46] study (48 hours). The 72 hours incubation period allows the activity of the substance to affect the merozoite reinvasion process whereas the 48 hours incubation period will only affect the intraerythrocytic growth of the malaria parasites. In this case, the MeOH extract of this plant might not so effective against the ring to schizont intraerythrocytic stages of *P. falciparum*. Other species of *Elephantopus* such as *E. mollis* have been shown to possess potential antiplasmodial activity (IC_50_ = 2.2 μg/ml) against CQ resistant *P. falciparum* (K1) [69].

### M. elliptica

To our knowledge, this plant had not yet been investigated for the *in vitro* antiplasmodial activity. However other species of *Morinda, M. morindoides* has been shown to exhibit a pronounced antiplasmodial activity [64].

### C. iners

The only antiplasmodial study on *C. iners* was previously reported for its roots extract. However, the MeOH extract of *C. iners* roots exhibited weak antiplasmodial activity (IC_50_ = 12.7 μg/ml) against CQ resistant *P. falciparum* (FCR-3) [21]. In contrast, the leaves extract (MeOH) of this plant which was prepared by present study exhibited a good antiplasmodial activity (EC_50_ = 0.63 μg/ml) (Table 4). In addition, other *Cinnamomun* species, *C. griffithii* has been reported to elicit a promising antiplasmodial activity (IC_50_ < 10 μg/ml) against both CQ sensitive and resistant *P. falciparum* strains [70]. To our knowledge, there is no compound related to this plant reported for antiplasmodial activity.

### Curcuma sp

The member of the *Curcuma* plant species such as *C. zedoaria*, *C. mangga*, *C. aeruginosa* and others were well studied for their antiparasiticidal properties [71]. The H_2_O extracts of *C. xanthorriza* and *C. aeruginosa* were previously found to be effective in inhibiting *P. falciparum in vitro* (40% and 90% inhibition, respectively). However the concentration of the extracts used (1 mg/ml) was too high [62]. Moreover, the major antiplasmodial compounds like curcumin and its derivatives such as demethoxycurcumin and bis-demethoxycurcumin isolated from *C. longa* exhibited high IC_50_ value (IC_50_ > 5 μM) [72]. In contrast, by different technique of plant extraction, the present study showed a promising antiplasmodial activity (EC_50_ < 10 μg/ml) of *C. zedoaria* (DCM extract), *C. aeruginosa* (H_2_O extract) and *C. mangga* (MeOH and H_2_O extracts) with negligible toxic effect on normal cell line (SI >10) (Table 4 and 5). Although the antiplasmodial activity of *Curcuma sp* isolated compound such as curcumin is considered weak, it was found to be very effective in antimalarial drug combination study [15, 73–75].

### V. amygdalina

The *V. amygdalina* plant is also found in African countries and is widely used traditionally in treating fever, malaria, measles, diabetes, worms, hypertension and others [76, 77]. The ethanol, petroleum ether, methylene chloride and MeOH extracts of *V. amygdalina* leaf have been previously reported to elicit IC_50_ values of less than 10 μg/ml against *P. falciparum* [64, 65]. Both ethanolic and H_2_O extracts of *V. amygdalina* leaf has also been shown to inhibit schizont maturation of fresh *P. falciparum* isolates from patients with negligible toxicity in rats [44]. In addition, the *in vivo* antimalarial activity of this plant has also been reported [66–68]. In *in vivo* drug combination experiment, the decoction of *V. amygdalina* leaves has the ability to enhance the CQ activity in *P. berghei* infected mice [68]. Furthermore, the infusion of *V. amygdalina* leaves has been clinically tested against the uncomplicated malaria where the parasite clearance has been documented [78]. So, it is not surprising to see a promising antiplasmodial activity of *V. amygdalina* leaf extracts (DCM, MeOH and H_2_O extracts) as showed by the present study (EC_50_ < 10 μg/ml) (Table 4). The antimalarial property of this plant could be due to the presence of its active constituents, sesquiterpene lactones such as vernolepin, vernolin, vernolide, vernodalin and hydroxyvernodalin [79].

### B. javanica

The *B. javanica* grows in Asia Pacific region including China, Indonesia, Malaysia and Thailand [45]. Different parts of this plant such as fruit, roots, seeds, stems and bark are traditionally used in treating variety of diseases including babesiosis, malaria and cancer. The H_2_O extract of *B. javanica* leaves, fruits and bark have been shown to possess a strong antiplasmodial activity against *P. falciparum* [62]. In the present study, the DCM and MeOH extracts of *B. javanica* leaves and roots showed good (EC_50_ < 1 μg/ml) to good to moderate (EC_50_ = 1.1 to 10 μg/ml) level of antiplasmodial activities against CQ resistant *P. falciparum*, K1 (Table 4). By similar extraction procedure, the DCM, MeOH and H_2_O extracts *B. javanica* roots have also been previously reported to possess a good to moderate level antiplasmodial activity against another CQ resistant *P. falciparum* strain, W2 with IC_50_ ranging from 1.0 to 2.0 μg/ml [51]. The antiplasmodial activity showed by this plant might be governed by its active constituents such as quassinoids, alkaloids (bruceacanthinoside) and triterpenoids (Bruceajavanin A and dihydrobruceajavanin A) [80, 81]. Other plant from the same family (*Simaroubaceae*) like *E. longifolia* also exhibited an antiplasmodial activity ruled by its quassinoids and alkaloids contents [25, 82]. In addition, the present study has identified the antiplasmodial activity from seeds of this plant (MeOH and H_2_O extracts). However, the DCM extract of the seeds seem to have weak or no antiplasmodial activity (EC_50_ > 15.7 μg/ml) (Table 4). In another study with different extraction procedure, the extracts of *B. javanica* fruits (ethanol, MeOH-ethanol, aqueous-MeOH residue, ethyl acetate and ethyl alcohol extracts) have been shown to have a promising antiplasmodial activity against *P. falciparum* K1 strain (IC_50_ < 10 μg/ml). In contrast, the H_2_O extract of the fruits showed weak antiplasmodial activity (IC_50_ > 10 μg/ml) [45].

### V. negundo

The *V. negundo* has been traditionally used in India as antiseptic, anti-inflammatory, antipyretic, treating enlargement of spleen and others [83]. The present study has highlighted the potential antiplasmodial activity of DCM (EC_50_ = 1.1 μg/ml) and MeOH (EC_50_ = 2.17 μg/ml) extracts of *V. negundo* leaves against the CQ resistant *P. falciparum*. The MeOH extracts of *V. negundo* leaf has been previously shown to exhibit a promising antiplasmodial activity against CQ sensitive (IC_50_ = 9.5 μg/ml) *P. falciparum* but not to CQ resistant *P. falciparum* strain (IC_50_ = 19.8 μg/ml). In similar study, the MeOH extract of the flower of this plant also exhibited a promising antiplasmodial activity with IC_50_ value of against CQ sensitive (IC_50_ = 2.8 μg/ml) but not to CQ resistant *P. falciparum* (IC_50_ = 17.8 μg/ml) [46].

### A. muricata

As reported by Osorio et al. [63], the hexane, ethyl acetate and MeOH extract of *A. muricata* leaf exhibited good to moderate level of antiplasmodial activities against CQ sensitive *P. falciparum* strain F32 (IC_50_ ranging from 7.2 to 9.2 μg/ml) but not to CQ resistant strain W2 (IC_50_ ranging from 10.4 to 38.6 μg/ml) [63]. However, the most potent ethyl acetate extracts of this plant is considered toxic to U-937 cells (human monocytes) (SI = 1.1 and 0.2 for F32 and W2 *P. falciparum* strains, respectively). On the other hand, with different extraction approach, the present study not only showing the promising antiplasmodial activity of *A. muricata* leaves extracts (DCM, MeOH and H_2_O extracts) (EC_50_ < 10 μg/ml), but also the non-toxic activity of the extract to MDBK cells (SI = 66-756) (Table 4 and 5).

## Conclusions

Twenty three extracts derived from *C. zedoaria* (rhizome), *C. aeruginosa* (rhizome), *A. galanga* (rhizome), *V. negundo* (leaf), *M. elliptica* (leaf), *C. mangga* (rhizome), *E. scaber* (leaf), *B. javanica* (leaf and root), *A. muricata* (leaf), *C. iners* (leaf) and *V. amygdalina* (leaf) showed the best antiplasmodial activities against the blood stage chloroquine resistant *P. falciparum* with no toxic effects on MDBK cells (SI ≥ 10). The present study has also scientifically supported the efficacy of *B. javanica* and *A. galanga* which are used traditionally to treat malaria in Peninsular Malaysia. Although these plant extracts were able to kill the *P. falciparum in vitro*, further *in vivo* evaluation is needed to demonstrate their efficacy in treating mammalian malaria model. Furthermore, the bioassay guided fractionation is a way forward for determination of bioactive compounds which will lead to the formulation of new antimalarial drugs or standardized antimalarial extracts.

## Authors’ information

All the authors are researchers from Bioassay and Phytochemistry Units of Herbal Medicine Research Centre, Institute for Medical Research, Kuala Lumpur, Malaysia.
